# Impact of *N*-Acetylation on DNA Damage and Oxidative Stress Responses in Mammalian Cells and Human Hepatocytes Treated with Hydralazine

**DOI:** 10.3390/biom16040562

**Published:** 2026-04-10

**Authors:** Mariam R. Habil, Makayla A. Stephens, Alexandra A. Cass, Elise M. Mittlestat, Darbie Kwon, Alexandra Ellison, J. Calvin Kouokam, David W. Hein

**Affiliations:** Department of Pharmacology and Toxicology, School of Medicine, University of Louisville, Louisville, KY 40202, USA; mariam.refaatzakyhabil@louisville.edu (M.R.H.); makayla.stephens@louisville.edu (M.A.S.); alexandra.cass@louisville.edu (A.A.C.); elise.mittlestat@louisville.edu (E.M.M.); darbie.kwon@louisville.edu (D.K.); alexandra.ellison@louisville.edu (A.E.); joseph.kouokam@louisville.edu (J.C.K.)

**Keywords:** hydralazine, human hepatocytes, DNA damage, γH2AX in-cell western assay, AP sites, antioxidant/ROS

## Abstract

Hydralazine is widely used to treat hypertension during pregnancy and has epigenetic effects in cancer therapy. Cryoplatable human hepatocytes showed concentration-dependent increase in DNA damage response (linear trend *p* = 0.0069) following 24 h hydralazine treatment. DNA repair-deficient UV5 Chinese hamster ovary (CHO) cell lines expressing human *CYP1A2* and either NAT2*4 (reference allele) or NAT2*5 (variant allele) were treated with hydralazine for 24 h. CHO cells expressing NAT2*4 showed a higher acetylation rate than those with NAT2*5 (*p* < 0.001), whereas CHO cell viability did not differ significantly following hydralazine treatment (*p* > 0.05). Hydralazine caused a concentration-dependent increase in DNA damage response in the un-transfected UV5 CHO cell line, as well as in each of the UV5 CHO cell lines transfected with human *CYP1A2* and/or *NAT2* alleles. CHO cells with *CYP1A2* only showed higher DNA damage response from hydralazine compared to cells with *CYP1A2*/NAT2*4 or *CYP1A2*/NAT2*5 (*p* < 0.05 and *p* < 0.0001, respectively), and higher in CYP1A2/NAT2*4 versus CYP1A2/NAT2*5 cells (*p* = 0.0011). Apurinic/apyrimidinic (AP) sites in CHO cells expressing only *CYP1A2* were significantly higher than in the un-transfected UV5 CHO cell line (*p* < 0.01) and higher in CHO cells expressing *CYP1A2*/NAT2*4 compared to *CYP1A2*/NAT2*5, but the difference was not significant (*p* > 0.05). In contrast, ROS levels were reduced following hydralazine treatment in CHO cells with *CYP1A2*/NAT2*4 and *CYP1A2*/NAT2*5 (*p* < 0.001 and *p* < 0.05, respectively). The results of the current study document DNA damage responses associated with hydralazine in human hepatocytes and CHO cells. The DNA damage response was increased following *N*-hydroxylation by CYP1A2, which competes with *N*-acetylation by NAT2.

## 1. Introduction

Arylamine *N*-acetyltransferase 2 (NAT2) is found on chromosome 8 at 8p22, spanning about 170–360 kb [1,2,3]. NAT2 exhibits polymorphism due to single-nucleotide polymorphisms (SNPs) that can affect substrate metabolism. This results in interindividual differences in both efficacy and toxicity following treatment with drugs such as hydralazine [4,5]. The reference allele NAT2*4 is linked to the rapid acetylator phenotype, while NAT2*5 is the most common slow acetylator allele [2]. It possesses two SNPs: 341T>C, I114T, rs1801280 and 481C>T, rs1799929. It was formerly identified as NAT2*5B, but now, it is identified as NAT2*5.002 [2].

Hydralazine constitutes a potent vasodilator broadly applied for the treatment of hypertension in pregnant women and heart failure cases [6]. Additionally, hydralazine plays an epigenetic role in cancer therapy by inducing reversible heritable changes in gene expression without altering the DNA sequence [7,8]. Hydralazine suppresses DNA methylation by downregulating the DNA methyltransferases that mediate aberrant cytosine methylation in mammalian DNA [9,10]. Clinical studies have reported an effective role of hydralazine, either alone or combined with valproate, in multiple malignancies, including cervical cancer [11] and hepatocellular carcinoma [12]. In addition, hydralazine is associated with a reduction in the incidence rate of hematologic neoplasms [13].

Two pathways for hydralazine metabolism in humans include direct acetylation via NAT2, producing 3-methyl-s-triazolo (3,4-a)-phthalazine (MTP), and production of *N*-acetyl hydrazinophthalazinone (NAc-HPZ) through its oxidative intermediate HPZ [14]. *N*-acetylation of hydralazine is NAT2 genotype-dependent in cryoplatable human hepatocytes [15]. Clinical studies revealed that blood pressure reduction following hydralazine treatment was greater in NAT2 slow acetylators versus NAT2 rapid acetylators [16]. NAT2 slow acetylators also are more susceptible to developing adverse effects such as drug-induced lupus erythematosus [17].

Previous studies have reported that hydralazine is mutagenic in bacteria [18,19]. Also, hepatocytes from rats [20] and slow NAT2 acetylator rabbits [21] showed DNA damage following treatment with hydralazine. Treatment of Chinese hamster ovary (CHO) cells with hydralazine led to chromosomal aberrations [22]. In addition, hydralazine formed covalent adducts in double- and single-stranded DNA, suggesting possible genotoxic effects [23]. However, the associations of *NAT2* allelic variants with hydralazine genotoxicity remain unclear. Additionally, more studies using human metabolic machinery are required for risk assessment of hydralazine. Therefore, this study aimed to investigate the effect of NAT2 on DNA damage and oxidative stress responses induced by hydralazine in human hepatocytes and CHO cells with human metabolic machinery.

## 2. Materials and Methods

### 2.1. Chemicals

Acetyl-coenzyme A, hydralazine hydrochloride, and 3-methyl-s-triazolo (3,4-a)-phthalazine (MTP) were purchased from Sigma Aldrich (St. Louis, MO, USA).

### 2.2. Source and Culture of Cryoplatable Human Hepatocytes

Cryoplatable human hepatocytes provided by BioIVT (Woodbury, NY, USA) (http://www.bioivt.com/) were stored in liquid nitrogen. The hepatocytes were obtained from donors providing consent under IRB-approved protocols at the FDA-licensed donor facility at BioIVT. They were isolated by BioIVT from freshly removed human transplant-rejected livers and frozen within a 24 h interval. All hepatocytes tested negative for hepatitis B and C, as well as HIV1 and HIV2. NAT2 genotypes and deduced phenotypes were determined as described previously [24]. Cryoplatable human hepatocytes were selected with intermediate acetylator genotypes, including 3 individuals with NAT2*4/*5 (n = 1, male) or NAT2*4/*6 (n = 2, 1 female and 1 male). For hepatocytes, INVITROGRO™ HI serum-free Medium (BioIVT, Woodbury, NY, USA) containing hydralazine (0–100 µM) was used for 24 h. Media were removed, and γH2AX in-cell Western staining was performed as previously described [24].

### 2.3. Construction and Characterization of UV5/Chinese Hamster Ovary (CHO) Cell Lines

UV5-CHO, a nucleotide excision repair (NER)-deficient cell line derived from AA8 cells [25], was provided by ATCC. Since UV5-CHO cells lack NER mechanisms because of a mutated XPD (ERCC2) gene, they are hypersensitive to bulky adduct mutagens. UV5-CHO cells harboring human *CYP1A2* and NAT2*4 or NAT2*5 were constructed as described in previous work [26]. Quantitative RT-PCR (RT-qPCR) was utilized to determine CYP1A2 mRNA levels in CHO cells, while CYP1A2 protein amounts were evaluated by in-cell western [24]. CHO cell lines underwent authentication using *NAT2* allele-specific polymerase chain reaction as outlined in a previous report [25]. This model lacks NER, making it more sensitive to genotoxins; therefore, it is difficult to compare the findings with those of human hepatocytes that presumably have a functional DNA repair system. Importantly, expressing *CYP1A2* with or without *NAT2* allowed investigating the effect of phase 1 and phase II metabolic pathways on hydralazine DNA damage.

### 2.4. N-Acetyltransferase Assays

*N*-acetyltransferase assays containing cell lysates of CHO cells expressing human *CYP1A2* and either NAT2*4 or NAT2*5, hydralazine (10 to 1000 µM) and acetyl-coenzyme A (300 to 1000 µM) underwent incubation at 37 °C for 1 h. The concentrations were selected based on preliminary experiments to generate a concentration–response curve. After terminating the reaction with 1/10 volume of 1 M acetic acid, the samples were submitted to a 10 min centrifugation at 13,000× *g* for protein precipitation. The acetylated product, 3-methyl-s-triazolo [3,4a]-phthalazine (MTP), was quantitated upon injection (40 µL) of each sample onto a 250 × 4 mm Discovery 5 µM C18 HPLC column. Hydralazine and MTP were separated with a gradient from 95:5 to 40:60 (55 mM sodium phosphate: methanol, pH 4) over 20 min, followed by a return to 95:5 over 3 min. This resulted in retention times of 8.9 and 19.9 min for hydralazine and MTP, respectively, with the UV detector set at 260 nm. Protein amounts of cell lysates were assessed with the Bio-Rad Bradford protein assay kit (Bio-Rad, Hercules, CA, USA), and enzyme activity was expressed in nmoles MTP/min/mg protein.

### 2.5. Cell Viability

The cytotoxic effect of hydralazine on CHO cells was assessed using the resazurin (Alamar Blue) cell viability assay (Thermo Fisher Scientific, Waltham, MA, USA) as directed by the manufacturer. Briefly, 1  ×  10^4^ cells were grown overnight in 96-well plates in alpha-modified minimal essential medium (MEM) (Cytiva, Logan, UT, USA) with L-glutamine, ribosides, and deoxy-ribosides supplemented with 10% fetal bovine serum (Hyclone, Logan, UT, USA), 100 units/mL penicillin, and 100 µg/mL streptomycin (Hyclone, Logan, UT, USA) at 37 °C in 5% CO_2_. Then, media containing hydralazine (0–100 µM) was added for 24 h. Next, the cells were rinsed with 1× PBS, and Alamar Blue solution (100 µL per well at a final concentration of 500 µM, dissolved in 1× PBS) was added for a 1 h incubation. Fluorescence was quantitated at excitation and emission wavelengths of 530 and 590 nm, respectively, on the Infinite 200 PRO microplate reader (Tecan, Männedorf, Switzerland).

### 2.6. DNA Damage Response

DNA damage response was assessed by the γH2AX in-cell western assay as outlined in a previous study [27]. In brief, 1  ×  10^4^ CHO cells were plated into black/clear bottom 96-well plates (Greiner Bio-One, Kremsmünster, Austria), or 5 × 10^4^ of cryoplatable human hepatocytes (with NAT2*4/5 or NAT2*4/6) were plated into black/clear bottom collagen coated 96-well plates (Corning^®^ BioCoat^®^, New York, NY, USA), and allowed to attach overnight. The next morning, medium was removed, and CHO cells were washed with PBS and replaced with fresh pre-warmed no-phenol-red MEM (FBS 5%) containing hydralazine (0–100 µM) and incubated for 24 h. For hepatocytes, INVITROGRO™ HI serum-free Medium (BioIVT, Woodbury, NY, USA) supplemented with TORPEDO antibiotic mix (BioIVT, Woodbury, NY, USA) containing hydralazine (0–100 µM) was used for 24 h. Media were removed, and the γH2AX in-cell western staining protocol was performed as previously reported [27].

### 2.7. DNA Extraction and Apurinic/Apyrimidinic (AP) Sites Measurement

CHO cells were seeded in 6-well plates at 5 × 10^5^ /well. After 24 h, the cells were briefly rinsed in phosphate-buffered saline (PBS) and incubated for 24 h in MEM media containing hydralazine (0–50 µM). Then, the cells were rinsed in PBS and collected by trypsinization, washed in PBS, and genomic DNA was extracted using the E.Z.N.A.^®^ Tissue DNA Kit (Omega Bio-tek, Norcross, GA, USA). Next, apurinic/apyrimidinic (AP) sites, which are common DNA lesions, were quantified using the DNA Damage Assay Kit (AP sites, Colorimetric; Abcam Inc., Waltham, MA, USA) according to the manufacturer’s instructions.

### 2.8. Intracellular ROS Detection with DCFDA

Intracellular ROS content was determined with 2′,7′-dichlorofluorescein diacetate (DCFDA) (Sigma-Aldrich, MO, USA) as in previous studies [27,28]. In brief, CHO cells underwent culture in the presence of selective agents in 100 mm Petri dishes. After harvest, 1  ×  10^4^ cells were seeded into black/clear bottom 96-well plates (Greiner Bio-One). The next morning, the medium was aspirated, and attached cells underwent washing with 1× PBS. Then, 20 mM of DCFDA (Sigma Aldrich, MO, USA) was prepared using DMSO, and then diluted to 5 µM in no-phenol-red α-Minimal Essential Medium (MEM), added to each well and incubated for 30 min at 37 °C. Then, cells were washed with hydralazine (0–50 µM) diluted in no-phenol-red α-Minimal Essential Medium, added and incubated for 30 min at 37 °C. Positive control wells were treated with H_2_O_2_ 1 mM for 30 min, and blank wells (with non-stained cells) were used as a control. The fluorescence intensity was measured using the Infinite 200 PRO microplate reader at Ex/Em. = 485/520 nm.

### 2.9. Statistical Analysis

Differences in *N*-acetylation rates were evaluated for significance by unpaired *t*-test. Cell viability, DNA damage, and ROS generation were evaluated for significance by two-way analysis of variance (ANOVA), followed by Bonferroni’s or Tukey’s post hoc test or an unpaired *t*-test. Statistical analysis was performed with GraphPad Prism 11 (GraphPad Software Inc., San Diego, CA, USA).

## 3. Results

### 3.1. Hydralazine Increases DNA Damage Response in Cryopreserved Human Hepatocytes

**Figure 1:**
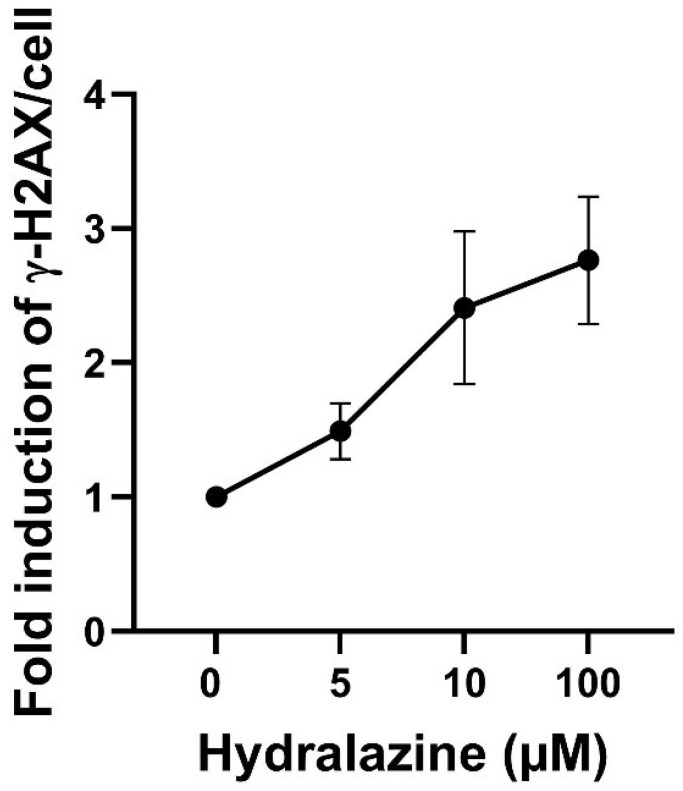
DNA damage response measured with the γH2AX in-cell western assay in cryoplatable human hepatocytes (NAT2*4/NAT2*5 or NAT2*4/NAT2*6 genotypes) treated with hydralazine (0–100 µM). Hydralazine treatment led to a significant (linear trend *p* = 0.0069) concentration-dependent increase in γH2AX signal. Statistical significance was determined using one-way ANOVA, followed by a linear trend test. Data represent mean ± SEM from three independent samples of cryoplataablee human hepatocytes.

Hydralazine treatment resulted in a significant (linear trend *p* = 0.0069) concentration-dependent increase in DNA damage response measured by γH2AX protein expression in cryopreserved human hepatocytes (Figure 1).

### 3.2. CHO Cells with Rapid NAT2 Acetylator Phenotype Show Higher Hydralazine N-Acetylation Rates Compared with Counterparts with Slow Acetylator Phenotype

**Figure 2:**
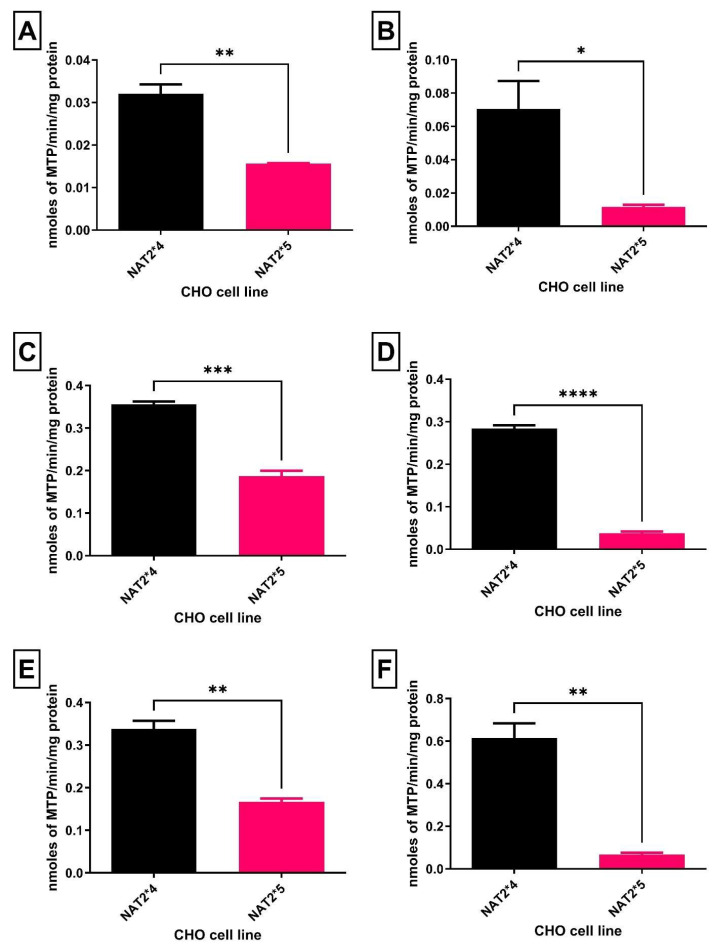
*N*-acetylation rates of hydralazine in vitro in CHO cells with AcCoA 300 µM (**left panel**) or 1000 µM (**right panel**). (Hydralazine 10 µM in (**A**,**D**). Hydralazine 100 µM in (**B**,**E**). Hydralazine 1000 µM in (**C**,**F**)). Statistical significance was determined using an unpaired *t*-test. Data represent mean ± SEM from three independent experiments. * = *p* < 0.05, ** = *p* < 0.01, *** = *p* < 0.001 **** = *p* < 0.0001.

Hydralazine *N*-acetyltransferase activities were determined in lysates obtained from UV5-CHO cells expressing human *CYP1A2* and NAT2*4 or NAT2*5 alleles. As shown in Figure 2, hydralazine NAT2 catalytic activity in CHO cells expressing NAT2*4 was about 2-fold higher than in the CHO cells expressing NAT2*5 with AcCoA concentrations of 300 µM and about 6 to 9-fold higher with AcCoA concentrations of 1000 µM.

### 3.3. Hydralazine Causes a Concentration-Dependent Increase in γH2AX Expression in CHO Cell Lines

**Figure 3:**
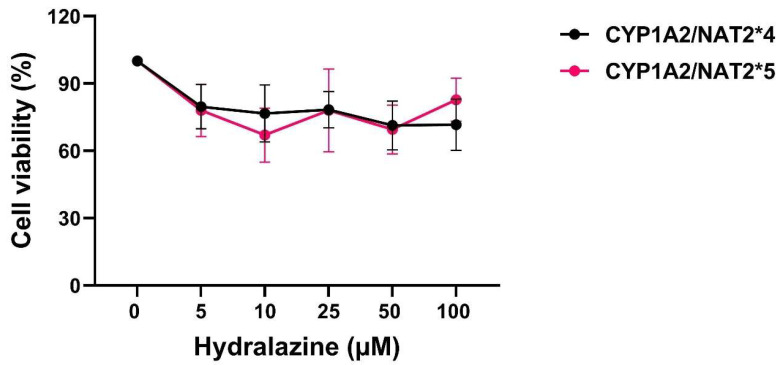
Cell viability measured with the Alamar blue assay in CHO cells treated with hydralazine (0–100 µM). No significant difference in cell viability was observed between CHO cells expressing NAT2*4 or NAT2*5 (*p* > 0.05). Data represent mean ± SEM from three or four independent experiments.

Before assessing DNA damage, cell viability was measured with the Alamar blue assay in CHO cells after treatment with hydralazine. As shown in Figure 3, no statistically significant difference was observed between CHO cells with NAT2*4 or NAT2*5.

**Figure 4:**
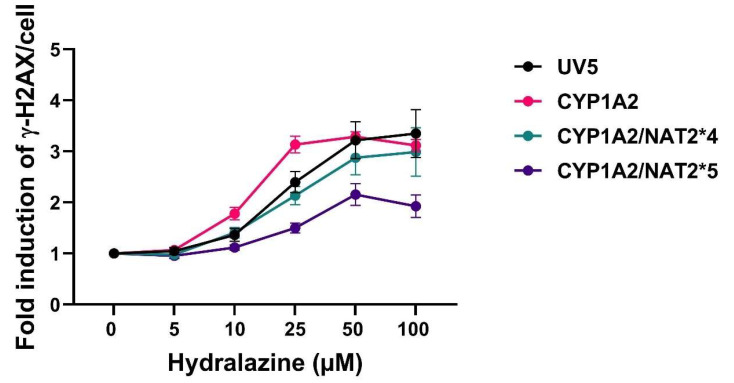
DNA damage response evaluated using the γH2AX in-cell western assay in CHO cells treated with hydralazine (0–100 µM). Statistical significance was determined using two-way ANOVA, followed by Tukey’s post hoc test. Data represent mean ± SEM from four independent experiments. The *CYP1A2* expression showed higher levels of DNA damage response than CYP1A2/NAT2*4 and CYP1A2/NAT2*5 expression (*p* < 0.05 and *p* < 0.0001, respectively). CYP1A2/NAT2*4 showed higher DNA damage response than CYP1A2/NAT2*5 (*p* = 0.0011).

DNA damage was examined by the γH2AX in-cell western assay that measures DNA damage response. Our data showed that hydralazine led to a concentration-dependent increase in γH2AX signal in all CHO cell lines. CHO cells expressing NAT2*4 showed higher levels of γH2AX signal than those expressing NAT2*5 (*p* = 0.0011), suggesting enhanced DNA damage response, as shown in Figure 4.

**Figure 5:**
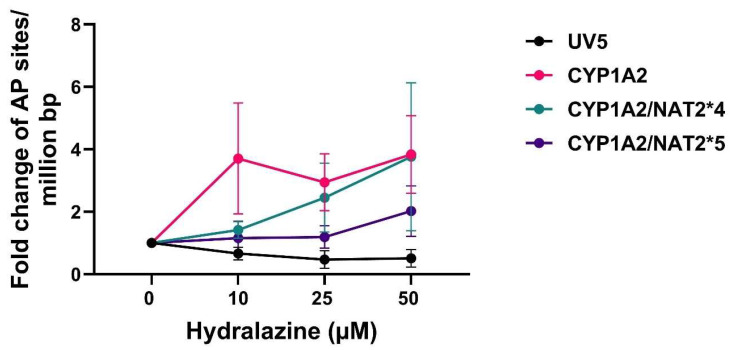
AP sites measured with the AP-DNA damage assay in CHO cells treated with hydralazine (0–50 µM). Statistical significance was determined using two-way ANOVA, followed by Tukey’s post hoc test. Data represent mean ± SEM from five independent experiments. AP site levels were significantly higher in CHO cells expressing *CYP1A2* than UV5 (*p* < 0.01). CHO cells expressing CYP1A2/NAT2*4 did not differ significantly (*p* > 0.05) from cells expressing CYP1A2/NAT2*5 or *CYP1A2* alone.

We further examined DNA damage by quantitating AP sites in the CHO cell lines. As shown in Figure 5, hydralazine treatment led to a decrease in AP sites in the un-transfected UV5 CHO cell line, whereas the other CHO cell lines expressing CYP1A2 and NAT2 showed an increase in AP sites after treatment with hydralazine. AP sites in CHO cells expressing only *CYP1A2* were significantly higher than in un-transfected UV5 (*p* < 0.01). AP sites were higher in CHO cells expressing NAT2*4 compared to NAT2*5, but the difference was not significant (*p* > 0.05).

### 3.4. ROS Levels in CHO Cell Lines Treated with Hydralazine

**Figure 6:**
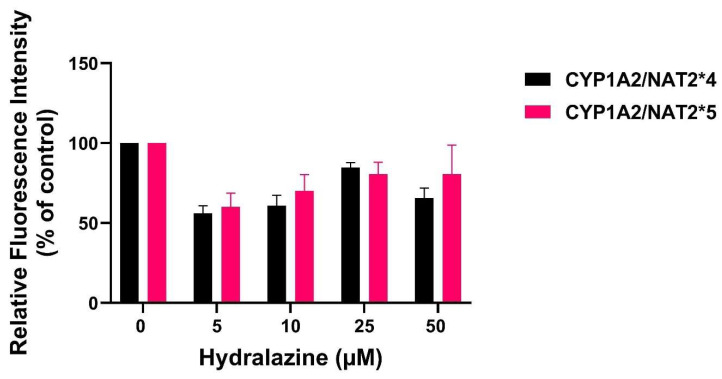
DCFDA assay in CHO cell lines expressing NAT2*4 or NAT2*5 following treatment with hydralazine (0–50 µM). ROS levels following hydralazine treatment were less than vehicle control with no significant difference in ROS level between CHO cells expressing *CYP1A2*/NAT2*4 and *CYP1A2*/NAT2*5 (*p* > 0.05). Data comparisons used two-way ANOVA with a Bonferroni post hoc test. Data represent mean ± SEM from four independent experiments.

Following treatment with hydralazine at concentrations up to 50 µM, as shown in Figure 6, ROS levels were less than vehicle control, with no significant difference in ROS level between CHO cells expressing NAT2*4 and NAT2*5 (*p* > 0.05).

## 4. Discussion

In the current study, cryoplatable human hepatocytes showed a concentration-dependent increase in DNA damage response following treatment with hydralazine. This is consistent with previous studies on human hepatocytes in which increases were reported in DNA fragmentation and DNA repair synthesis [20]. Previous studies reported hydralazine DNA damage response in hepatocytes isolated from slow NAT2 acetylator rabbits but not from rapid NAT2 acetylator rabbits [21]. However, the rates of *N*-acetyltransferase activity measured in the human hepatocytes did not result in meaningful effects on the DNA damage endpoints [20]. The objective of the present study was to investigate the effects of *N*-acetylation of hydralazine in CHO cells expressing human NAT2*4 and NAT2*5 to evaluate the hypothesis that *N*-acetylation rates of hydralazine affect DNA damage and oxidative stress responses.

Significant differences were observed in *N*-acetylation rates of the CHO cells expressing human NAT2. The highest levels were observed in cells with NAT2*4, and lower levels were found in cells with NAT2*5. This is consistent with previous studies on bacteria, where NAT overexpressing *Salmonella* strain YG1029 produced over five times higher levels of *N*-acetylated metabolite than *Salmonella* TA100, which is a normal NAT expressor strain [29].

As for cell viability, a previous study reported that the IC50 of hydralazine was 243.3 ± 16.9 µg/mL, corresponding to approximately 1.2 mM [22], which is higher than the concentration range used in the current study (0–100 µM). We found similar levels of cell viability between CHO cells expressing CYP1A2/NAT2*4 and CYP1A2/NAT2*5 without marked cell toxicity (approximately 30% cytotoxicity maximum).

DNA damage induced by hydralazine was previously reported in different bacterial and mammalian models [19]. Our findings on CHO cells and cryopreserved human hepatocytes document concentration-dependent DNA damage responses, as measured by γH2AX protein expression following treatment with hydralazine. CHO cells expressing *CYP1A2/NAT2* reduced the DNA damage response, as measured by γH2AX protein expression, compared to CHO cells expressing *CYP1A2* alone.

To investigate other mechanisms of DNA damage induced by hydralazine, we measured AP sites formation, which revealed lower levels of AP sites in UV5 cells compared with other cell lines, especially *CYP1A2*-transfected cells. Other CHO cell lines had an increasing trend in AP sites formation after hydralazine treatment, suggesting that human metabolic enzymes are required to generate this form of DNA damage. AP sites formation is a well-known mechanism of action for currently used chemotherapeutic agents such as alkylating agents [30,31]. Recently, Islam et al. reported that hydralazine inhibited APE1, the enzyme required for AP sites repair in cell-free systems [32]. We measured the intracellular formation of AP sites directly following treatment of CHO cells with hydralazine. This supports the efficacy of hydralazine as a chemotherapeutic based on its effect on increasing AP sites, which are toxic to cancer cells.

Our results showed reduced ROS generation following treatment with hydralazine, suggesting that it might have an antioxidant role, consistent with previous studies that reported the protective role of hydralazine against ROS production in normal and cancer cell lines [33,34,35].

Our findings are consistent with the anticancer actions of hydralazine. Human leukemic T-cells Jurkat showed an increase in γH2AX protein expression following treatment with hydralazine, which was associated with apoptosis [9]. Further studies reported the antioxidant effect of hydralazine in normal and cancer cell lines [33,34,35,36], providing another mechanism of the anticancer effect of hydralazine that aligns with our current findings showing that hydralazine decreased ROS generation. The DNA damage response may be mediated indirectly via oxidative stress induced by hydroxylation products of hydralazine catalyzed by CYP1A2. However, measurements of other oxidative stress parameters, like MDA for lipid peroxidation or superoxide dismutase as an antioxidant, are needed to confirm the findings.

The previously reported effects of metabolic pathways on DNA damage induced by hydralazine are inconsistent. The previous studies reported on bacteria where mutagenicity from hydralazine was lower in *Salmonella* strain YG1029, which overexpresses NAT, than in TA100 and TA100/1,6DNP *Salmonella* strains, which express normal or lack NAT protein expression, respectively [29]. Another study done on rabbit hepatocytes reported that hydralazine increased DNA repair synthesis, indicative of DNA damage, which resulted from hydralazine treatment in hepatocytes isolated from slow NAT2 but not rapid NAT2 acetylator rabbits [21]. Although a previous human hepatocytes study reported that NAT2 measured with sulfamethazine did not result in meaningful effects on DNA damage response [20], it is noted that the study measured DNA fragmentation and DNA repair synthesis in primary cultures of hepatocytes from four human donors, and information on *NAT2* genotype was not provided.

## 5. Conclusions

In the current study, we found a concentration-dependent increase in DNA damage response measured by γH2AX in human hepatocytes and both γH2AX and AP site formation in the CHO cell lines expressing human metabolic enzymes. CHO cells with *CYP1A2* only showed the highest levels of both γH2AX and AP sites formation following hydralazine treatment, suggesting that the DNA damage response was increased following *N*-hydroxylation by CYP1A2, which competes with *N*-acetylation by NAT2. Additional studies using human hepatocytes from rapid and slow NAT2 acetylators are necessary to further investigate the role of rapid and slow NAT2 acetylator phenotypes. Taking all together, our study provides valuable information for hydralazine hazard identification and risk assessment as an anti-hypertensive agent.

## Data Availability

The original contributions presented in this study are included in the article. Further inquiries can be directed to the corresponding author.

## Abbreviations

*N*-acetyltransferase 2 (NAT2); acetyl-coenzyme A (AcCoA); single nucleotide polymorphisms (SNPs); metabolite 3-methyl-s-triazolo (3,4-a)-phthalazine (MTP); *N*-acetylhydrazinophthalazinone (NAc-HPZ); hydrazinophthalazinone (HPZ); Chinese Hamster Ovary (CHO); alpha-modified minimal essential medium (MEM); and apurinic/apyrimidinic (AP); phosphate-buffered saline (PBS); analysis of variance (ANOVA); 2′,7′-dichlorofluorescein diacetate (DCFDA).
